# Additively Manufactured Electrodes for Simultaneous Detection of 2,4,6-Trinitrotoluene and Cyclotrimethylenetrinitramine in Postexplosive Residues and Environmental Samples

**DOI:** 10.1021/acsomega.5c02191

**Published:** 2025-06-11

**Authors:** Gilvana P. Siqueira, Raquel G. Rocha, Mariana C. Marra, Mario H. P. Santana, Eduardo M. Richter, Rodrigo A. A. Muñoz

**Affiliations:** a Institute of Chemistry, 425927 Federal University of Uberlândia, Uberlândia, Minas Gerais 38408-100, Brazil; b Forensic Laboratory of the Federal Police, Uberlândia, Minas Gerais 38408-663, Brazil

## Abstract

This work reports the use of a custom-made filament based on polylactic acid and graphite to construct additively manufactured working electrodes, using fused filament fabrication 3D-printing technology, to simultaneously detect the explosives 2,4,6-trinitrotoluene (TNT) and cyclotrimethylenetrinitramine (RDX). We propose a simple strategy to increase detectability that consists of an electrochemical preconcentration step (−1.3 V *vs* Ag|AgCl|KCl_(sat.)_ for 30 s) to reduce RDX and TNT species on the 3D-printed electrode prior a single differential pulse stripping voltammetry scan in Britton–Robinson buffer (pH = 6.0). Linear ranges of 2.5–30 μmol L^–1^ and 50–500 μmol L^–1^ and limit of detection (LOD) values of 0.57 and 8.67 μmol L^–1^ were achieved for TNT and RDX, respectively. With this approach, it was possible to identify TNT and RDX in seawater and post-explosive samples. Recovery values (80–99%) for both compounds in tap water without a sample dilution step achieved through the standard addition method, attested the high performance of proposed method. This work highlights that 3D-printing technology is a powerful tool to construct portability and system for determining nitrocompounds in environmental and forensic applications.

## Graphical abstract

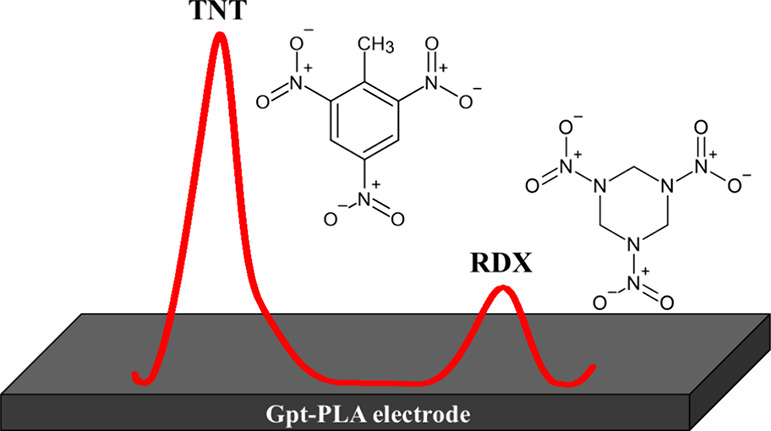

## Introduction

Nitroaromatic compounds are a critical class of substances used primarily as precursors in the manufacture of explosive devices. These compounds have diverse applications across various fields, including road construction, mining, engineering, space science (for rocket propulsion), and military operations.^1^ Generally, explosives such as 2,4,6-trinitrotoluene (TNT) and cyclotrimethylenetrinitramine (RDX) are used for this purpose. In forensic scenarios, the prevalence of explosives has notably increased in criminal incidents such as terrorist attacks and bank robberies. For example, 2521 people died in terrorist attacks involving bomb explosions around the world in 2021. Additionally, between 2017 and 2018, 3214 banks in Brazil were targeted with explosives.^2^

The growth of military exercises, such as those conducted at training sites and on battlefields, along with other activities like mining and quarrying, releases harmful chemicals into the environment, leading to pollution of both marine and terrestrial ecosystems.^3^ Furthermore, nitroexplosives used during the two World Wars remain widespread in the environment even decades later.^3,4^ It is also important to highlight that nitroaromatic compounds are persistent environmental pollutants, largely due to their inherent chemical structure.

Characterized by the presence of three nitro groups attached to an aromatic ring, these compounds are highly electron-deficient and demonstrate notable environmental persistence.^5^ Their specific spatial arrangement contributes to the longevity of residues from explosions and unexploded ordinance, which can persist in the environment for extended periods. This prolonged persistence leads to significant toxic and polluting effects.^6^ For example, RDX exposure can cause problems such as convulsions, vertigo, and vomiting, which has even been reported from the US and European factory employees.^7^ Moreover, long TNT exposure can potentially harm the natural environment, including marine and coastal life and marine and coastal lives. Research indicates that TNT and its post-explosion products can damage the spleen and liver and may have mutagenic and carcinogenic effects.^8^

Different analytical techniques have been applied to determine trace levels of explosives, such as Raman and infrared (IR),^9^ capillary electrophoresis,^10^ and gas chromatography–mass spectrometry;^11^ however, they are time-consuming and costly and require qualified personnel to operate and interpret the results. In this way, electrochemical methods are a powerful portable tool to detect harmful chemicals (such as explosives), due to their properties, including rapid response, appropriate selectivity, and sensitivity, wide linear range, and low-cost instrumentation.^12^ Research on the electrochemical detection of nitroaromatic species at trace levels has been conducted using different working electrodes substrates, such as graphene,^13^ carbon black,^14^ reduced graphene oxide,^15^ gold nanoparticles,^16^ graphite sheets,^17^ and the combination of carbon nanotubes and gold nanoparticles.^18^

The advent of additive manufacturing, particularly fused deposition modeling (FDM) also known as fused filament fabrication (FFF), has received significant interest from researchers due to its properties, such as ability for rapid lab-prototyping with customizable designs and shapes, as well as lower waste generation compared to traditional subtractive manufacturing processes, resulting in reduced costs. Various filaments, such as commercially available conductive fillers based on carbon black (CB) and polylactic acid (PLA), as well as homemade alternatives, have been utilized to create working electrodes.^19,20^ Many research groups have proposed electrochemical methods using 3D-printed electrodes to determine many explosives.^21–24^ Despite the abundance of studies involving commercial filaments, electrodes produced from these materials often exhibit poor electrochemical performance in their native form (as-printed), in which requires additional surface activation.^20,25^

To address this issue, recent efforts have focused on increasing the concentration of conductive fillers within composite filaments to enhance the electrochemical activity of additively manufactured electrodes (AMEs).^26^ Stefano and co-workers^27^ developed a filament containing 40% wt graphite (Gpt) within a matrix of PLA and, remarkably, presented a superior voltammetric profile for the redox probe ferro/ferricyanide compared to commercially available AMEs, without the need for any activation steps.

Although electrochemical methods seem to be important tools for detecting explosive residues, challenges remain for the simultaneous determination of these compounds. This is because the voltammetric signals produced by these electrochemical methods often result in complex, overlapping voltammograms with multiple peaks. Moreover, the selective detection of TNT and RDX is important because many types of explosives are combinations of both compounds, including Torpex A (45% TNT, 37% RDX, and 18% aluminum powder) and Baratol Comp. B-2 (60% TNT, 40% RDX), among others. Recently, Castro et al. showed that it is possible to detect both substances using cyclic square-wave stripping voltammetry (CSWSV) on graphite electrodes. In this method, TNT is detected through its electrochemical reduction (cathodic scan) after an accumulation step at 0.0 V for 15 s, and RDX is detected in the reverse scan via the oxidation of its reduction products generated at −0.9 V (anodic scan).^17^

Herein, we first proposed the simultaneous determination of RDX and TNT in a single scan using AMEs made from graphite (40 wt %) and PLA (60% wt.). Notably, 3D-printed working electrodes require only surface polishing to achieve a smooth surface, which is an important property in the 3D-printing field, as most additively manufactured electrodes require additional surface treatment protocols.

## Experimental Section

### Chemical and Solutions

All reagents were used without further purification and were analytical grade. TNT and RDX were donated by Brazilian Federal Police (Uberlândia, Brazil). Boric acid was acquired from AppliChem Panreac (Barcelona, Spain). Phosphoric and acetic acids from Vetec (Rio de Janeiro, Brazil). Ferrocenemethanol (FcMeOH, 97% w/w), urea (99% w/w), and graphite powder (<20 μm) were obtained from Sigma-Aldrich (St Louis, USA). Acetonitrile was purchased from Êxodo Cientifica (São Paulo, Brazil). PLA pellets (without any pigments) were acquired from 3D LAB (Belo Horizonte, Brazil). Chloroform and acetone from Dinamica (São Paulo, Brazil). Sodium hydroxide was obtained from ChemiFlex (São Paulo, Brazil). Nitrobenzene (NB) (98% w/w), sodium nitrite (97% w/w), and hydrogen peroxide (29.0% w/v) were obtained from Synth (Diadema, Brazil). Sodium nitrate (99% w/w) from Merck (Darmstadt, Germany). Ammonium chloride (99% w/w) from Cinetica Química (Itapevi, SP, Brazil). Pentaerythritol Tetranitrate (PETN) was obtained from the Military Police of Minas Gerais (Belo Horizonte, Brazil). Picric acid in acetonitrile (PA) was obtained from Sigma-Aldrich (St. Louis, USA).

Britton–Robinson buffer (pH = 2.0 to 12.0) was prepared by mixing equimolar concentrations of acetic, phosphoric, and boric acids (0.04 mol L^–1^). A solution of 1 mol L^–1^ NaOH solution was used to adjust the pH. The stock solutions of TNT and RDX were prepared daily by dissolving appropriate amounts in acetonitrile (20 mmol L^–1^) and subsequently diluting in the corresponding supporting electrolyte. All stock aqueous solutions were prepared using high purity deionized water (resistivity≤ 18.2 MΩ) from a Millipore Direct-Q3 water purification system (Bedford, MA, USA).

### Sample Preparation

To evaluate the applicability of the proposed method, recovery experiments were carried out in environmental (seawater and tap water) and forensic (TNT explosion simulation) samples. Tap water samples were collected in our laboratory (Uberlândia, Brazil). Seawater was collected at Praia da Avenida Litorânea (São Luís, Brazil). For environmental samples, the sample preparation step involved dissolving the necessary reagents directly in the water samples to prepare the supporting electrolyte. After this, the water samples were spiked with different concentrations of TNT and RDX, and the concentrations were determined using the standard addition method.

TNT explosion simulation was performed with the assistance of the Brazilian Federal Police in Uberlândia (Minas Gerais, Brazil). The simulation was performed on a metal surface using TNT paste and a detonator mixture of lead azide and pentaerythritol tetranitrate. After the explosion, a cotton swab was used to sample the residual dust on the blast surface. Each swab underwent a simple extraction process, where 1 mL of supporting electrolyte was added, followed by vortex agitation for 2 min to extract the TNT residues.

### Preparation of Filament and Additively Manufactured Electrode

The composite filament made from graphite and PLA (Gpt-PLA) was synthesized using a solvent method, as previously described by Stefano and co-workers.^27^ Briefly, 20 g of graphite powder was dispersed in a mixture of acetone and chloroform (3:1 v/v), under magnetic stirring and reflux at constant temperature, 70 °C, for 30 min. After that, 30 g of PLA pellets was then added to the mixture and kept under constant stirring and heating for more than 3 h. The composite material obtained was recrystallized in ethanol, washed several times, with same solvent, and dried at 50 °C for 24 h. Subsequently, the composite was cut into small pieces and placed in a Filmaq 3D Extruder (Curitiba, Brazil) at 220 °C to obtain a filament with a 1.75 mm diameter. This filament was used to construct a 3D-printed square-shaped (measuring 11 mm in length, 11 mm in width, and 2 mm in thickness) working electrode, using a Flashforge Dreamer NX 3D printer (São José dos Campos, Brazil) with the following parameters: a printing perimeter speed of 30 mm s^–1^, two perimeters, a layer thickness of 0.05 mm, and 0.8 mm heated nozzle at 220 °C, as recommended in a previous work.^28^

### Instrumentation

All electrochemical measurements (cyclic voltammetry (CV), and differential pulse (DPV) voltammetry) were conducted using a μAutolab III potentiostat/galvanostat (Metrohm Autolab BV, Utrecht, Netherlands) with NOVA 2.1.7 software for data acquisition and processing, including baseline correction for DPV scans. All experiments were performed at room temperature (25 °C) in the presence of dissolved oxygen. A platinum wire and an Ag|AgCl saturated with 3.0 mol L^–1^ KCl were employed as counter and reference, respectively. For the simultaneous detection of RDX and TNT, the potential of −1.3 V (*vs* Ag|AgCl|KCl_(sat.)_) was first applied for 30 s to accumulate the reduction products of both species. Next, an anodic DPV scan was performed using optimized parameters (modulation time = 40 ms, amplitude = 100 mV, and step potential = 5 mV), and specific voltammetric peaks were registered for each explosive. A 3D-printed electrochemical cell (volume = 10 mL) was printed using polyethylene terephthalate glycol (PETG) filament (GTMax 3D, São Paulo, Brazil). More details about the construction of this cell were presented in a previous publication.^29^ A rubber O-ring delimited the geometric area (0.19 cm^2^). Figure S1 shows real images of the 3D-printed electrochemical cells and AMEs used in this work.

## Results and Discussion

### Electrochemical Behavior of RDX and TNT at Gpt-PLA Electrodes

As previously reported,^27,30^ working electrodes fabricated from lab-made graphite and PLA exhibit enhanced electrochemical activity compared to other additively manufactured electrodes (AMEs), requiring only mechanical polishing to smooth the electrode surface. Stefano and co-workers systematically evaluated graphite-PLA (Gpt-PLA) electrodes and, through Raman spectroscopy, confirmed the presence of D, G, and 2D bands. The D and G vibrational bands are associated with structural defects such as sp^3^ and sp^2^ carbon networks, vacancies, edge sites, or heteroatoms. According to the authors, the intensity of these bands increases after mechanical polishing, which, in addition to smoothing the surface, also promotes the exposure of conductive material. Scanning electron microscopy (SEM) images obtained by Siqueira et al.^30^ revealed the presence of flakes embedded within the PLA matrix, attributed to the incorporation of graphite in the composite.

Electrochemical characterization was also performed, including cyclic voltammetry—which revealed increased current intensity and reduced peak-to-peak separation for FcMeOH—and electrochemical impedance spectroscopy (EIS), which demonstrated a lower charge transfer resistance when compared to AMEs fabricated from commercial filaments.^27,30^

Subsequently, Gpt-PLA surfaces were evaluated through CV scan studies (from 10 to 200 mV s^–1^), using 1 mmol L^–1^ FcMeOH in 0.1 mol L^–1^ KCl solution, as shown in Figure S6. These results allow the estimation of the electroactive area, using the Randles–Sevcik equation:^31^

$$I_{p=}\pm 0.446n\mathrm{FAC}\sqrt{\frac{nFD\nu }{RT}}$$

where *n* is the number of electrons, *F* is the Faraday’s constant, *D* is the diffusion coefficient (FcMeOH: 7.6 × 10^–6^ cm^2^ s^–1^), *R* is the universal gas constant, *T* is the temperature, ν is the CV scan rate, and *A* is the electroactive area. Thus, the electroactive areas before and after mechanical polishing were estimated using the data presented in Figure S2. The electroactive areas were 0.15 and 0.16 cm^2^ before and after mechanical polishing, respectively. As observed, the electroactive area estimated in these experiments were similar probably due to the Randles–Sevick equation is time scale-dependent.^32^ Thus, the electroactive area in typical CV conditions is close to geometric area.^32,33^

Recently, our research group systematically investigated the TNT electrochemical behavior at Gpt-PLA electrodes to enable its detection in environmental samples.^30^ Siqueira et al. showed that CV measurements in the presence of 1 mmol L^–1^ TNT in 0.01 mol L^–1^ HCl solution revealed three reduction processes at approximately −0.23, −0.36, and −0.54 V (*vs* Ag|AgCl|KCl_(sat.)_) and a redox pair between +0.1 and +0.6 V (*vs* Ag|AgCl|KCl_(sat.)_). The electrochemical reduction reactions of TNT occur in three steps of six electrons each, related to the reduction of the nitro groups in TNT structure to hydroxylamine, followed by a reduction step to an amine group.^34^ Additionally, in the second scan, a redox pair is related to hydroxylamine electrochemical reaction.^35^ The electrochemical reaction of TNT is shown in Figure S3.

To the best of our knowledge, the RDX detection at 3D-printed carbon-based PLA electrodes has not yet been evaluated. In this context, the electrochemical performance of 1 mmol L^–1^ RDX was assessed at different pH values (2.0–10.0), using BR buffer (Figure S4). The results revealed a single reduction process at around −1.0 V (*vs* Ag|AgCl|KCl_(sat.)_) related to the reduction of the nitro group to *N*-nitroso groups.^36^ The only reduction peak observed for RDX corresponds to the stepwise reduction of the nitro group, initially to a nitroso intermediate, and subsequently to an amine at more negative potentials.^37^ In the reverse scan, an oxidative signal at around +0.9 V (*vs* Ag|AgCl|KCl_(sat.)_) was observed, which is attributed to the oxidation of the reduced species to a nitro group.^38^ As expected, a decrease of the RDX signal in a basic medium is observed due to the degradation of this compound under alkaline conditions.^39^ Additionally, the first CV cycle of the cathodic scan (−1.0 V *vs* Ag|AgCl|KCl_(sat.)_) displayed an irreversible reduction peak (R1), while the oxidation process (O1) only appeared in the second scan, as seen in Figure S5. This result suggests that the O1 peak at around +0.9 V, observed only in the second cycle, is dependent on the reduction process that occurred in the first scan (R1) as previously reported by Galik and co-workers^38^ using screen-printed carbon-based electrodes. Figure S6 presents the electrochemical reaction for RDX detection.

Given that the electrochemical reaction of TNT occurs readily across all pH ranges,^30^ the optimal conditions for simultaneous detection of nitro compounds were chosen by selecting the medium that resulted in the highest response for RDX and the most effective peak separation of oxidation processes for both molecules. Consequently, the BR buffer at pH 6.0 was chosen for further investigation.

Figure 1A presents a comparison of CVs scans obtained in the presence of RDX (blue line) and TNT (red line) in BR buffer (pH = 6.0) as a supporting electrolyte. The data reveal three distinct reduction peaks for TNT at −0.43, −0.62, and −0.79 V (*vs* Ag|AgCl|KCl_(sat.)_), while RDX exhibits a single reduction peak at −0.93 V (*vs* Ag|AgCl|KCl_(sat.)_). This difference in peak profiles reflects the higher energy required for the reduction of nitramines, such as RDX, compared to nitroaromatics such as TNT. The aromatic ring in TNT allows for delocalization of the negative charge, facilitating its reduction, whereas the aliphatic nature of RDX does not offer similar charge delocalization.^38^

**Figure 1:**
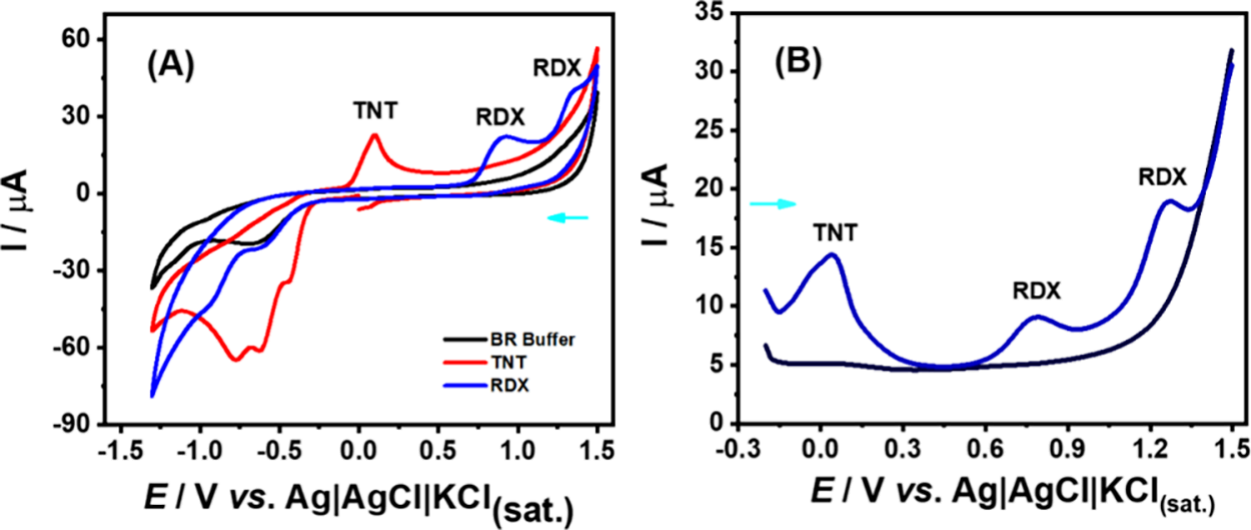
(A) Cyclic voltammograms recorded before (black line) and after the addition of 1 mmol L^–1^ RDX (blue line) or 0.1 mmol L^–1^ TNT (red line). The scan was performed from 0.0 to −1.3 V; −1.3 to +1.5 V; +1.5 to 0.0 V. CV conditions: scan rate = 50 mV s^–1^ and step potential = 5 mV. (B) DPSV obtained in the presence both nitrocompounds (25 μmol L^–1^ TNT and 200 μmol L^–1^ RDX) after applying a preconcentration potential step (−1.3 V/30 s). Other DPSV conditions: modulation time = 40 ms; amplitude = 100 mV; step potential = 5 mV. Other conditions: working electrode: Gpt-PLA. Supporting electrolyte: BR buffer (pH 6.0).

It is important to highlight that the reduction peaks of both molecules overlap; thus, it is difficult to discriminate them in different matrix samples (Figure S3). However, in the anodic scan, TNT undergoes oxidation in a single step (at around +0.02 V), whereas RDX displays two distinct oxidation processes at +0.71 and +1.13 V (*vs* Ag|AgCl|KCl_(sat.)_). These oxidation peaks are separated by 217 mV, which clearly allows for differentiation between the evaluated nitrocompounds. In this sense, a simple strategy was proposed to determine simultaneously TNT and RDX in the same sample (Figures 1B). To enhance the detection of nitrocompounds, the differential pulse stripping voltammetric technique was employed. This approach involved the reduction of nitrocompounds and the preconcentration of their reduced forms on the electrode surface, followed by a stripping step to detect the electrochemical oxidation of these reduced species.

Initially, preconcentration was achieved by applying various potentials ranging from −0.9 to −1.5 V for 60 s, using 25 μmol L^–1^ TNT and 200 μmol L^–1^ RDX solutions. The responses of TNT and RDX were assessed based on their oxidation peaks (see Figure S8A,B). It was observed that the electrochemical response for both compounds increased with the application of −1.3 V (*vs* Ag|AgCl|KCl_(sat.)_). Higher standard deviations were noted at potentials more negative than −1.4 V (*vs* Ag|AgCl|KCl_(sat.)_), possibly due to hydrogen evolution. Thus, −1.3 V (*vs* Ag|AgCl|KCl_(sat.)_) was selected as the optimal preconcentration potential for further studies. In the next step, the deposition time was also studied (5–180 s), and the best electrochemical response (current intensity) for both compounds, along with the appropriate analytical frequency, was achieved at a deposition time of 30 s.

After that, the DPV parameters (modulation time, amplitude, and step potential) were systematically optimized (unvaried test) based on sensitivity, speed of analysis, and peak shape, using 25 μmol L^–1^ TNT and 200 μmol L^–1^ RDX in BR buffer (pH = 6.0). Figure S9A–F exhibits the voltammograms obtained in this study. Better responses for both nitrocompounds were assessed using the following parameters: modulation time = 40 ms, step potential = 5 mV, and modulation amplitude = 100 mV. Indeed, the DPV scans outlined in Figure 1B displayed that both molecules are clearly detected with this strategy.

Under the optimized conditions, calibration curves were constructed separately for TNT (Figure 2A) and RDX (Figure 2C). Figure 2B shows the linear DPV response (2.5 to 30 μmol L^–1^) for TNT detection (*I* (μA) = 0.364 ± 0.006 *C*_TNT_ (μmol L^–1^) + 0.076 ± 0.069, *R* ^2^ = 0.998). On the other hand, it can be observed that the first oxidation peak increases linearly with RDX concentration within a wide linear range (50–500 μmol L^–1^) following this equation: *I* (μA) = 0.009 ± 0.001*C*_RDX_ (μmol L^–1^) – 0.341 ± 0.026; *R* ^2^ = 0.999 (Figure 2D). It is important to note that due to the unstable nature of the second oxidation process of RDX, we chose to use the first oxidation peak for quantification purposes. The limit of detection (LOD) was determined by using the IUPAC recommendation (3σ/*s*), where σ represents the standard deviation of the intercept of the calibration curve and *s* is the slope of the curve (sensitivity). The LOD values obtained were 0.57 and 8.67 μmol L^–1^ for TNT and RDX, respectively. A summary of the results obtained is shown in Table 1.

**Figure 2:**
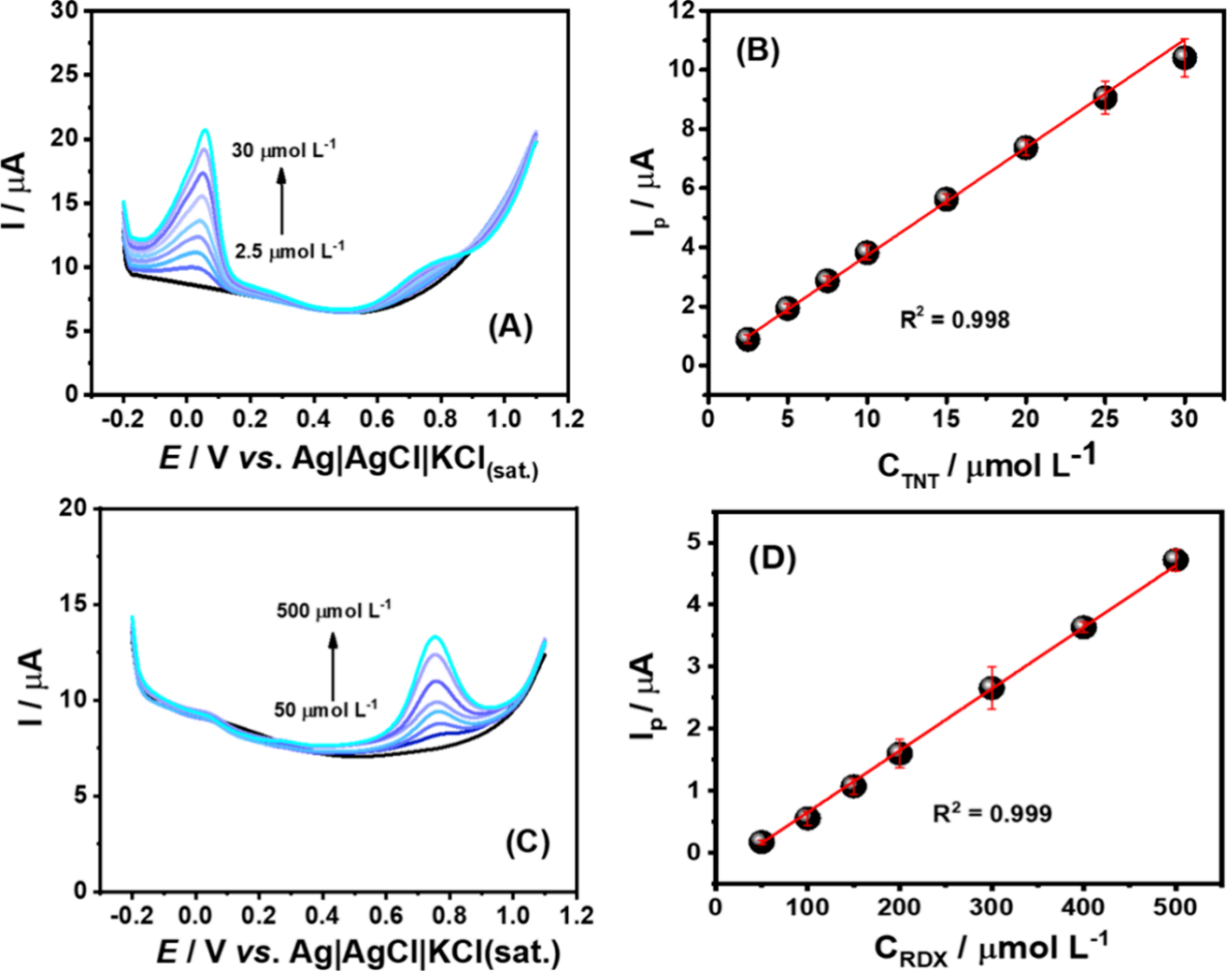
Baseline-corrected DPSV scans obtained in BR buffer (pH = 6.0) for increasing concentrations of (A) TNT (2.5–30.0 μmol L^–1^) and (C) RDX (50–500 μmol L^–1^) with (B, D) respective calibration plots, using Gpt-PLA electrodes. An application of −1.3 V/30 s was used for preconcentration. DPV conditions: modulation time = 40 ms; step potential = 5 mV; amplitude = 100 mV.

**Table 1:** Analytical Parameters Obtained for TNT and RDX Determination using 3D-Printed Gpt-PLA Electrodes

**analytical parameters**	**TNT**	**RDX**
linear range (μmol L^–1^)	2.5–30.0	50.0–500.0
*R* ^2^	0.998	0.999
intercept (μmol L^–1^)	0.076 ± 0.069	–0.341 ± 0.026
slope (μA L μmol^–1^)	0.364 ± 0.006	0.009 ± 0.001
LOD (μmol L^–1^)	0.57	8.67
LOQ (μmol L^–1^)	1.89	28.89
RSD (intra-eletrode, *n* = 10; 20 μmol L^–1^ TNT and 100 μmol L^–1^ RDX) (%)	2.0	3.1
RSD (inter-electrode, *n* = 3; 20 μmol L^–1^ TNT and 100 μmol L^–1^ RDX) (%)	2.4	4.0

It is important to mention that the difference in the estimated LOD and the sensitivity for both compounds can be associated to their structures.^16^ As previously reported, nitramines such as RDX are known to be more challenging to electrochemically react than nitroaromatics (TNT) due to their aliphatic nature. Unlike nitroaromatics, which benefit from the delocalization of negative charge around an aromatic ring, nitramines lack this stabilization mechanism, making their electrochemical reduction more difficult.^16,38^

To determine simultaneously RDX and TNT, calibration curves were performed by using the proposed method. First, RDX concentration was fixed (200 μmol L^–1^) and the concentration of TNT was increased in the electrochemical cell (2.5–30.0 μmol L^–1^) (Figure S10A,B). A wide linear range was achieved in this experiment; however, a slight reduction of sensitivity (slope = 0.189 μA L μmol^–1^) was achieved. Additionally, the increase in TNT did not significantly decrease the current intensity of RDX. In the next step, TNT concentration was fixed at 15 μmol L^–1^ and the concentration of RDX was varied (50.0–500.0 μmol L^–1^, slope = 0.031 μA L μmol^–1^). As observed in Figure S6, the higher RDX concentration resulted in a reduction of the TNT current intensity, probably due to the competition to occupy the electrode active sites.

The intraelectrode precision of the proposed method was evaluated using consecutive differential pulse voltammetry (DPV) scans (*n* = 10) in the presence of 20 μmol L^–1^ TNT and 100 μmol L^–1^ RDX (Figure S11). The relative standard deviation (RSD) values were below 3.1%, demonstrating good repeatability of the method. Interelectrode precision was also assessed at the same concentration levels using three different electrodes (*n* = 3), as shown in Figure S12. The RSD of peak current intensities for both explosives was below 4.0%, indicating good accuracy and reproducibility of the proposed method.

### Comparison of the Proposed Electrochemical Method with Other Reported in the Literature

The analytical performance of the proposed method was compared with other electrochemical sensors reported in the literature for RDX and/or TNT detection, as outlined in Table 2. Although many works reported in the literature presented superior analytical performance to detect RDX or TNT, most of them did not present simultaneous detection of studied nitrocompounds or present some interference of nitrocompounds. For example, Alizadeh and co-workers^40^ constructed a molecularly imprinted polymer (MIP) on multiwalled carbon nanotube and glassy carbon electrode to determine RDX in environmental samples; however, the authors observed an interference of TNT and 1,3,5,7-tetranitro-1,3,5,7-tetrazocane (HMX) in the electrochemical response of RDX. Additionally, the construction of these sensors involves laborious and costly modifications steps.

**Table 2:** Comparison of Analytical Parameters (LOD and Linear Range) Obtained for Some Electrochemical Sensors Reported in the Literature for the Detection of RDX or TNT^a^

**analyte**	**technique**	**electrode**	**linear range** **(μmol L** ^**–1**^ **)**	**LOD** **(μmol L** ^**–1**^ **)**	**detection of other nitrocompounds**	**ref**
RDX	DPV	MIP/MWCNTs-GC	0.01–10.0	0.01	No	^40^
	CSWSV	GS	20–300	2.4	TNT	^17^
	CV- DWT and ANN	SPCE	NR	NR	TNT and PETN	^41^
	SWAdsV	GCE	280–580	45	No	^44^
	DPSV	Gpt-PLA	50–500	8.67	TNT	this work
TNT	CSWSV	GS	1–150	0.1	RDX	^17^
	CV	P(*o*-PDA-*co*-ANI)-AuNPs/GCE	11–176	9.25	No	^45^
	DPV	G Na_2_SO_4_/GCE and G LiClO_4_/GCE	17.6–88	17.0 and 8.9	No	^13^
	DPV	B:DGNW	0.2–8.8	0.32	No	^46^
	DPV	multilayer graphene nanoribbons	NR	4.4	No	^47^
	DPSV	Gpt-PLA	2.5–30.0	0.57	RDX	this work

a Technique: DPV, differential pulse voltammetry; CSWSV, cyclic square-wave stripping voltammetry; SWAdsV, square-wave adsorptive voltammetry; DPSV, differential pulse stripping voltammetry; CV, cyclic voltammetry; DWT, discrete wavelet transform; ANN, artificial neural network model. Electrode: B:DGNW, boron-doped diamond/graphene nanowall electrodes; GCE, glassy carbon electrodes; GS, graphite sheet; MIP/MWCNTs-GC, molecularly imprinted polymer nanosphere/multiwalled carbon nanotube-coated glassy carbon electrode; SPCE, screen-printed carbon electrode; G Na_2_SO_4_/GCE or G LiClO_4_/GCE, anodic exfoliation of graphite foil in different electrolytes (LiClO_4_ and Na2SO_4_) on glassy carbon electrode; P­(*o*-PDA-*co*-ANI)-AuNPs/GCE, gold nanoparticles/poly­(*o*-phenylenediamine–aniline) film-modified glassy carbon electrode. NR, reported.

Cetó et al.^41^ advanced the field by demonstrating a method for the simultaneous detection of TNT, RDX, and pentaerythritol tetranitrate (PETN) in explosive samples, using chemometric data treatment of CV data. However, the application of this technique for on-site analysis is challenging due to the need for specialized personnel to interpret the results.

It is important to emphasize that 3D-printing technology represents a significant advancement in the development of electrochemical sensors, offering reduced costs while maintaining appropriate selectivity and sensitivity. Despite some studies have reported the use of 3D-printed electrodes for detecting various nitro explosives,^21,42,43^ these approaches often involve activation procedures to enhance the electrochemical activity of the sensors. In contrast, our methodology employs additively manufactured electrodes made from lab-made filaments, which did not require additional surface treatments. This approach enhances the practicality and effectiveness of the on-site analysis.

### Electrochemical Determination of RDX and TNT in Water and Postexplosion

TNT detection was evaluated in the presence of postexplosion residues (see Sample Preparation in the Experimental Section), as shown in Figure 3. A well-defined oxidation peak corresponding to TNT (dark blue lines) was observed in all samples. This was further confirmed by adding 20 μmol L^–1^ of TNT to the sample (light blue lines), with no significant changes in the peak potential of the target molecule were observed. Additionally, the introduction of 100 μmol L^–1^ of RDX (red lines) resulted in well-defined RDX peaks, demonstrating the sensor’s ability to detect RDX residues in postexplosion samples. These findings emphasize the sensor’s strong potential for detecting explosive residues containing both analytes.

**Figure 3:**
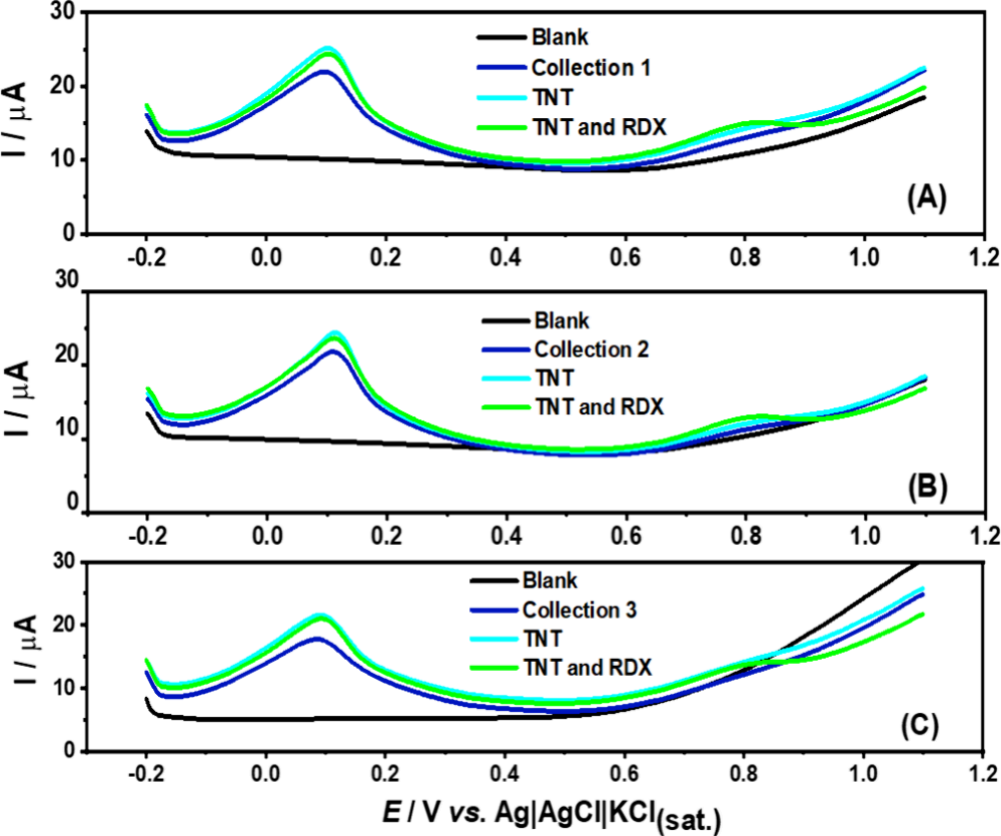
Differential pulse stripping voltammetry (DPSV) measurements were performed on Gpt-PLA for the TNT residues after (A) Collection 1, (B) 2, and (C) 3 (dark blue lines). Cyan lines represent the addition of 20 μmol L^–1^ of TNT, while green lines correspond to the subsequent addition of 100 μmol L^–1^ of RDX. The black lines refer only to the blank signal (BR buffer, pH 6.0). Application of −1.3 V/30 s to pre-concentrate reduction products. DPV conditions: modulation time = 40 ms; step potential = 5 mV; amplitude = 100 mV.

The applicability of the method was also evaluated for the detection and quantification of TNT and RDX in environmental samples, as shown in Figure 4. The simultaneous detection of TNT and RDX in seawater samples was successfully achieved, as demonstrated by the results in Figure S13. Furthermore, TNT and RDX were successfully quantified in tap water samples using the standard addition method. Recovery values ranging from 80 to 99% confirm the accuracy of the proposed method in this matrix, as presented in Table 3.

**Figure 4:**
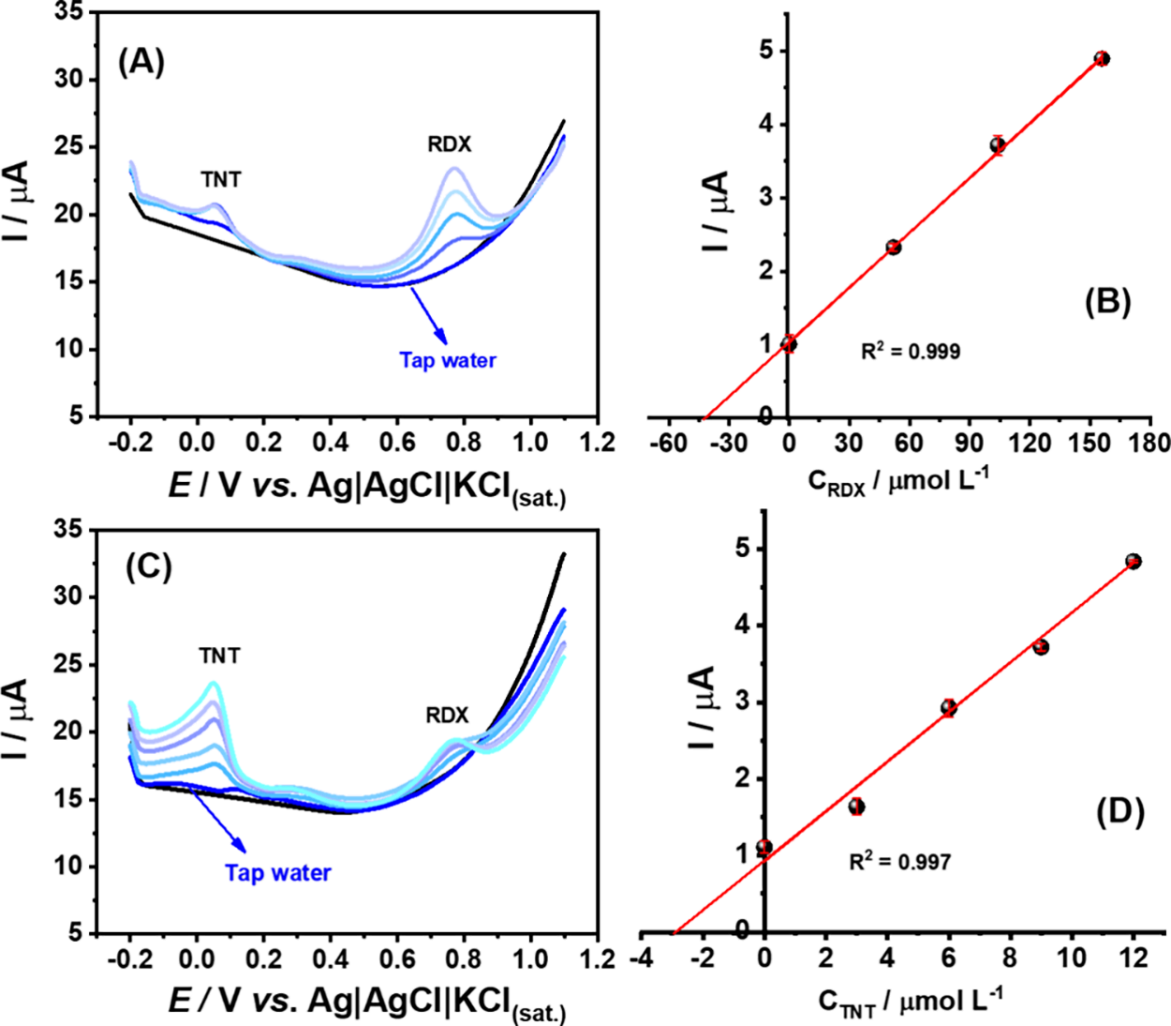
DPSV measurements were performed on Gpt-PLA for the proposed analysis of a tap water sample (dark blue lines). (A) The sample was spiked with 52 μmol L^–1^ of RDX (cyan line), followed by increasing additions of 52 μmol L^–1^ of RDX, while maintaining the TNT concentration at 5 μmol L^–1^. (B) Corresponding calibration curve for RDX obtained by standard addition. (C) The sample was spiked with 3 μmol L^–1^ TNT (cyan line), followed by increasing the additions of 3 μmol L^–1^ TNT, while maintaining the RDX concentration at 60 μmol L^–1^. (D) Corresponding calibration curve for TNT obtained by standard addition. The black lines refer only to the blank signal (BR buffer, pH 6.0). An application of −1.3 V/30 s to preconcentrate reduction products. DPV conditions: modulation time = 40 ms; step potential = 5 mV; amplitude = 100 mV.

**Table 3:** Determination of TNT and RDX in Tap Water Samples using the Gpt-PLA Electrode^a^

analyte	spiked (μmol L^–1^)	found (μmol L^–1^)	recovery (%)
TNT	3.0	3.0 (±0.2)	99 (±9)
RDX	52.0	40.3 (±2.7)	80 (±6)

a Values determined are the average response of three measurements for each sample.

### Interfering Species

The response of TNT and RDX was evaluated in the presence of potential interfering species, commonly found in explosive residues, as shown in Figure 5. It can be observed that the addition of these interfering species did not affect the detection peak potentials of TNT and RDX. Regarding its impact on the observed peak currents, a variation of less than 2.5% was recorded for TNT and 8.4% for RDX in the presence of 50 μmol L^–1^ of PETN. However, when 50 μmol L^–1^ NB was present, a more significant effect on the RDX peak current was observed, resulting in a reduction of approximately 25.6 and about 5.6% for TNT. In the presence of 50 μmol L^–1^ PA, both TNT and RDX peak currents decreased, with RSD values of 23 and 14%, respectively. Importantly, in all cases, the detection and quantification of TNT and RDX remained fully achievable.

**Figure 5:**
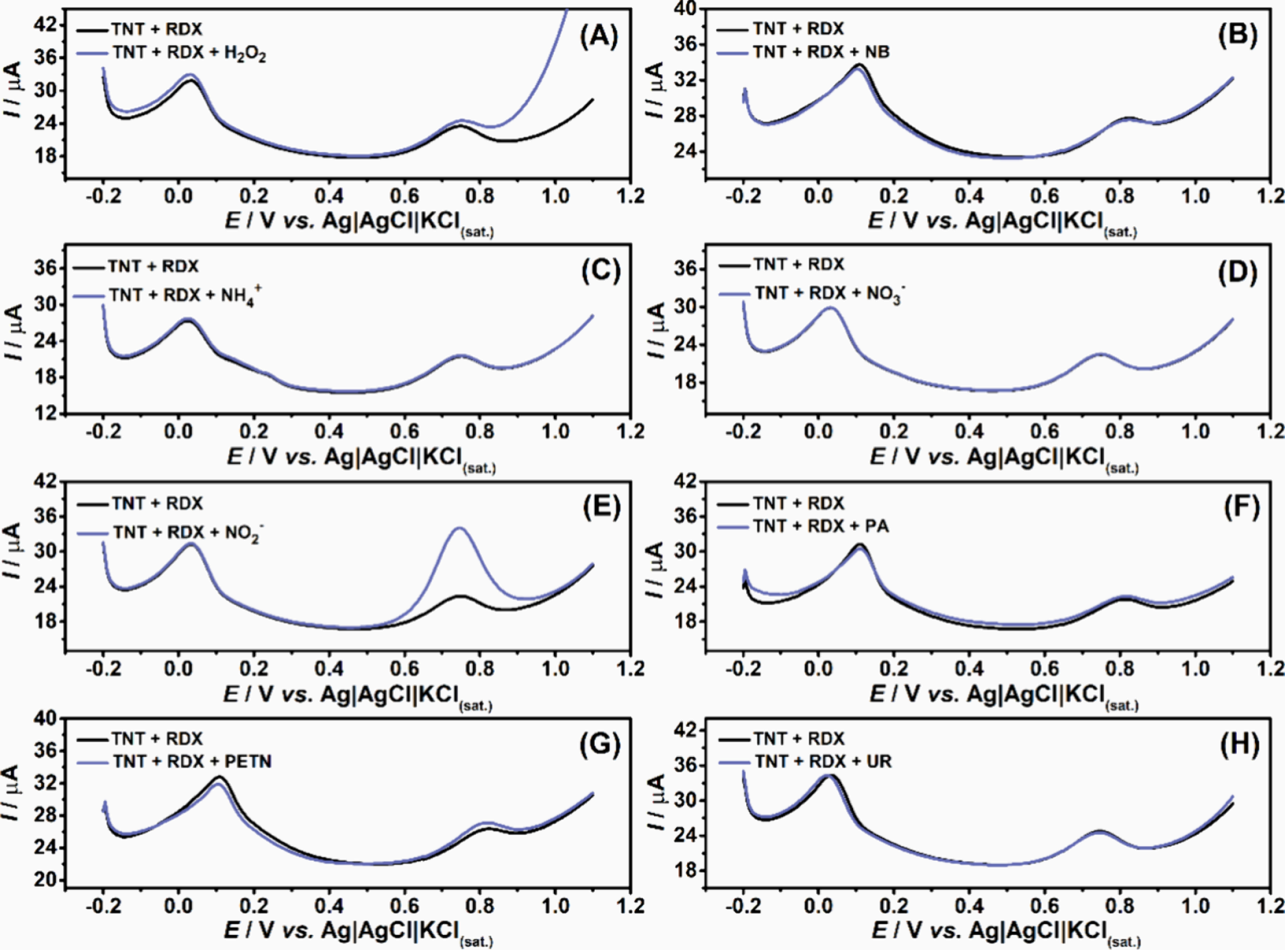
DPV measurements performed in the presence of 20 μmol L^–1^ TNT and 100 μmol L^–1^ RDX (black lines) and in the presence of 50 μmol L^–1^ of potential interfering species (blue lines), including (A) H_2_O_2_, (B) NB, (C) NH_4_^+^, (D) NO_3_^–^, (E) NO_2_^–^, (F) PA, (G) PETN, and (H) UR. An application of −1.3 V/30 s to preconcentrate reduction products. DPV conditions: modulation time = 40 ms; step potential = 5 mV; amplitude = 100 mV.

Other species commonly found in water samples, such as H_2_O_2_, NH_4_^+^, urea (UR), NO_2_^–^, and NO_3_^–^, each at 50 μmol L^–1^, were also evaluated (Figure 5). As observed, H_2_O_2_, NH_4_^+^, UR, and NO_3_^–^ did not interfere with the electrochemical detection of TNT and RDX. However, NO_2_^–^ significantly affected the electrochemical response of RDX. Therefore, a sample preparation step is required to remove NO_2_^–^ prior to analysis.

## Conclusions

Herein, we demonstrated that both TNT and RDX can be detected in a single scan, using a simple strategy to preconcentrate their reduced products through an application potential of −1.3 V (vs Ag|AgCl|KCl_(sat.)_) for 30 s, using Gpt-PLA as working electrodes. The proposed analytical method is free from interference of some explosives, including PETN. The proposed method was successfully applied to detect TNT and RDX in postexplosives and environmental (seawater and tap water) samples.

Importantly, 3D-printed electrodes made from lab-made conductive filament did not require any modification or treatment on the surface to determine both nitrocompounds. This approach enables routine analysis mainly for on-site forensic applications. In addition, additive manufacturing offers key advantages, including versatility and high sensitivity with a lower cost than conventional electrodes.

## Supplementary Material



## Notes

pH effect (Figure S1); oxidation study (Figure S2); supporting electrolyte effect (Figure S3); preconcentration study (Figure S4); DPV parameter optimization (Figure S5); calibration curves for TNT and RDX (Figure S6); detection of TNT and RDX in seawater samples (Figure S7) (PDF)
