# Supplementary material for: Additively Manufactured Electrodes for Simultaneous Detection of 2,4,6-Trinitrotoluene and Cyclotrimethylenetrinitramine in Postexplosive Residues and Environmental Samples
Source: ACS Omega. 2025 Jun 11;10(29):31577–86. doi: 10.1021/acsomega.5c02191 (PMC12311659; doi:10.1021/acsomega.5c02191)
Supplement: Supplementary file 1 [file ao5c02191_si_001.pdf]

Supporting information

Additively manufactured electrodes for simultaneous detection of TNT and RDX in post-explosive residues and environmental samples

Gilvana P. Siqueira[a], Raquel G. Rocha[a*], Mariana C. Marra[a], Mario H. P. Santana, Eduardo M. Richter[a], Rodrigo A. A. Muñoz[a*]

[a]Institute of Chemistry, Federal University of Uberlândia, 38408-100, Uberlândia, Minas Gerais, Brazil

[b]Forensic laboratory of the Federal Police, 38408-663, Uberlândia, Minas Gerais, Brazil.

Corresponding author:

[*]*munoz@ufu.br*

*raquel_rocha1793@hotmail.com

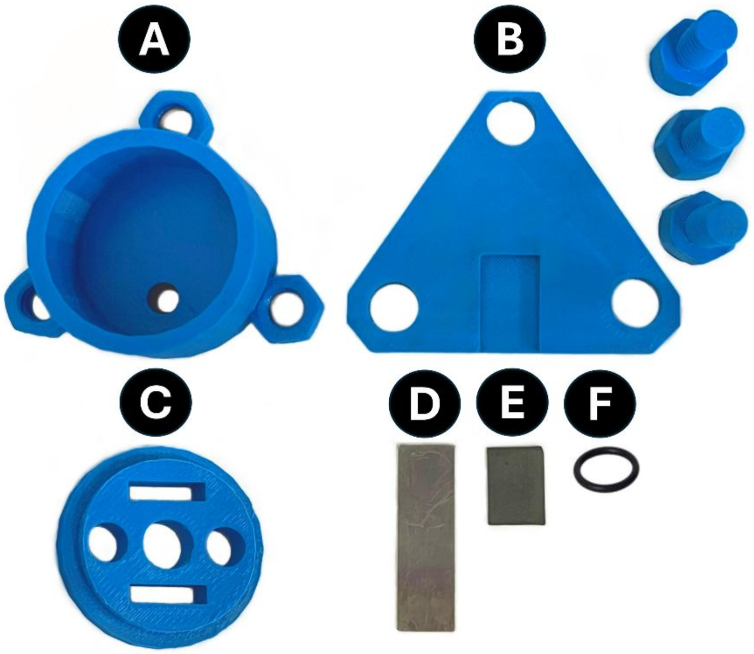

Figure S1. Images of the components of the 3D-printed electrochemical cell: **(A)** cell body; **(B)** bottom cover and three screws; **(C)** top cover; **(D)** steel plate; **(E)** working electrode; **(F)** O-ring. Adapted with permission from Carvalho, Mayane S.; Rocha, Raquel G.; de Faria, Lucas V.; Richter, Eduardo M.; Dantas, Luiza M. F.; da Silva, Iranaldo S.; Muñoz, Rodrigo A. A. Additively manufactured electrodes for the electrochemical detection of hydroxychloroquine. **Talanta**, 2022. 123727. Copyright 2025, with permission from Elsevier.

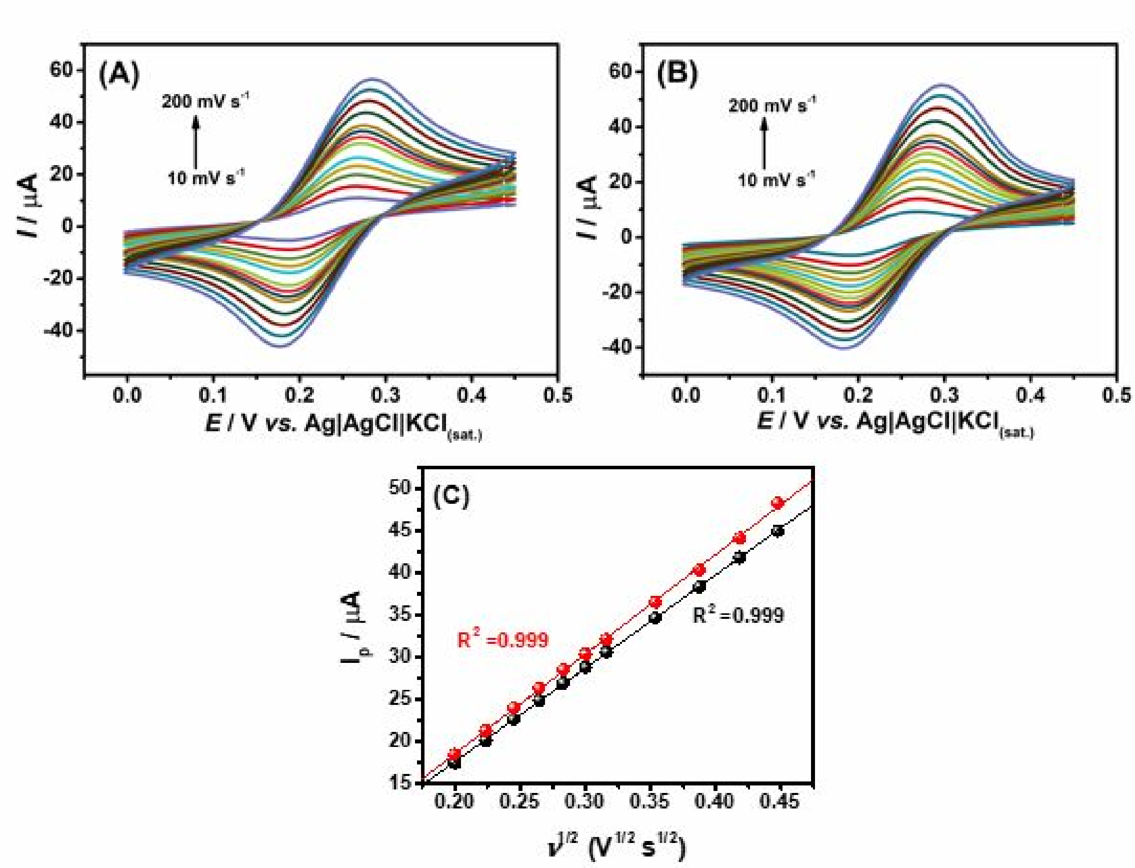

Figure S2. Cyclic voltammograms recorded at scan rates from 10 to 200 mV s^{-1} in the presence of 1.0 mmol L^{-1} ferrocenemethanol in 0.1 mol L^{-1} KCl at unpolished **(A)** and polished **(B)** Gpt-PLA electrodes. (C) plot of anodic peak currents (Ipa) versus the square root of the scan rate: unpolished (black dots) and polished (red dots) Gpt-PLA electrodes

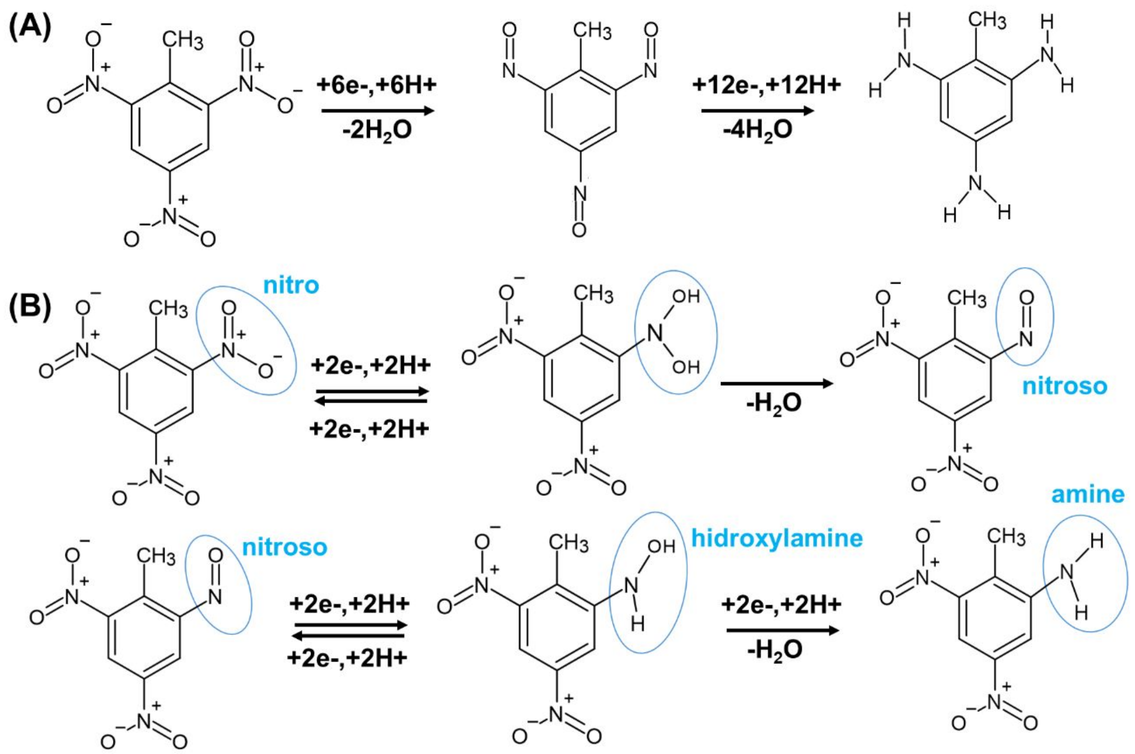

Figure S3. Electrochemical reaction of TNT: **(A)** reduction of all three nitro groups, and **(B)** reduction of a single nitro moiety in aqueous solution.

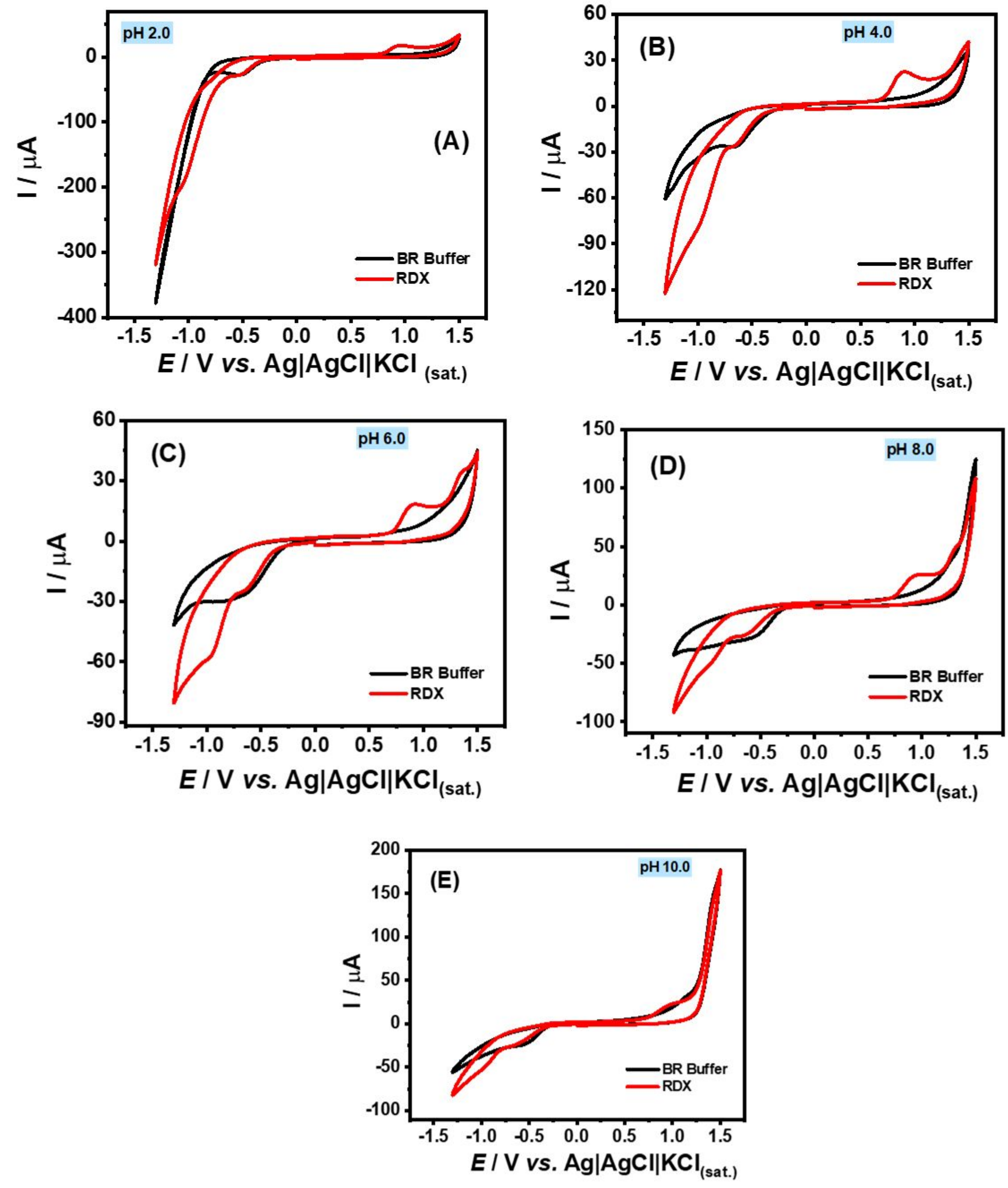

Figure S4. Cyclic voltammograms recorded in the presence of 1 mmol L^{-1} RDX, using BR buffer as supporting electrolyte at different pH values (2.0-10.0). Conditions: step potential: -5 mV, $v = 50$ mV s^{-1}.

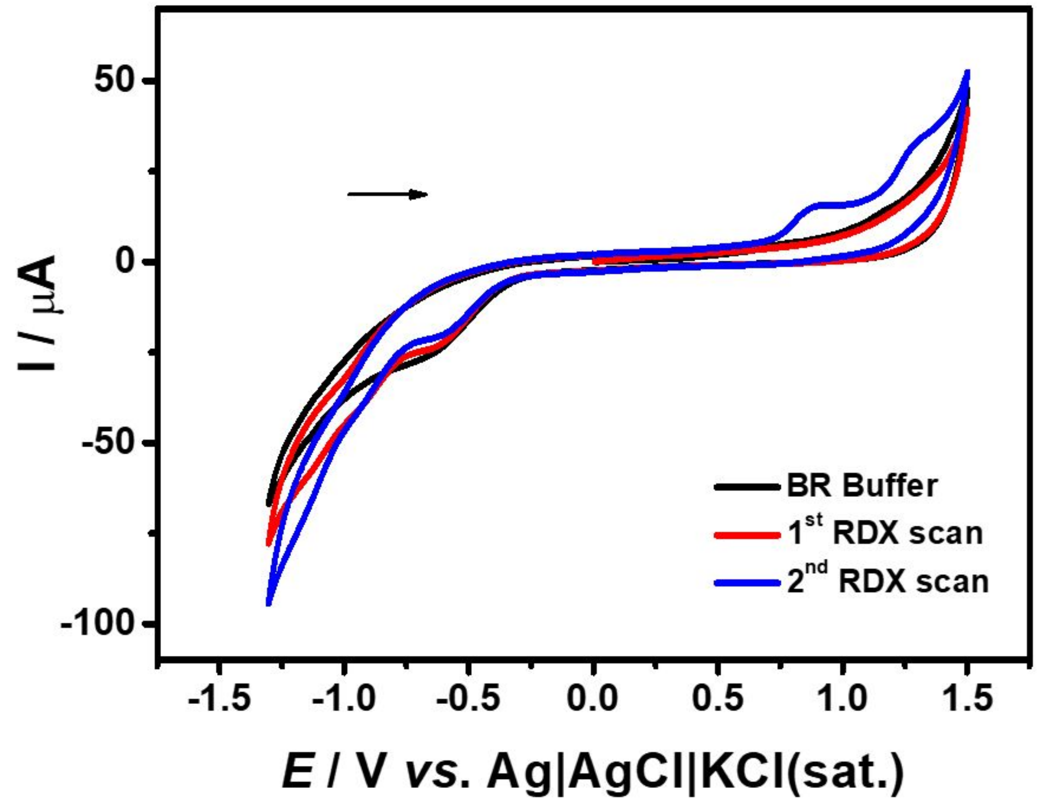

Figure S5. Cyclic voltammograms of the 1st and 2nd scans, recorded in BR buffer (pH 6.0); in the presence of 1 mmol L^{-1} RDX. Conditions: step: 5 mV, v = 50 mV s^{-1}.

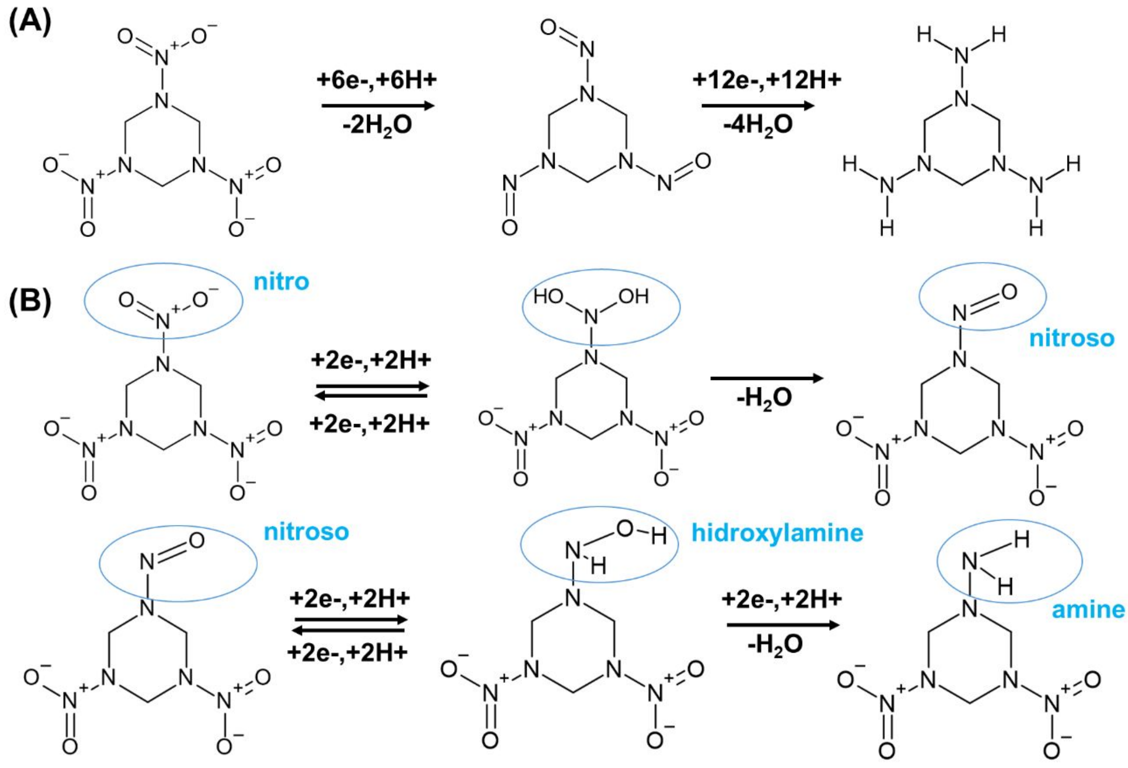

Figure S6. Electrochemical reaction of RDX: **(A)** reduction of all three nitro groups, and **(B)** reduction of a single nitro moiety in aqueous solution.

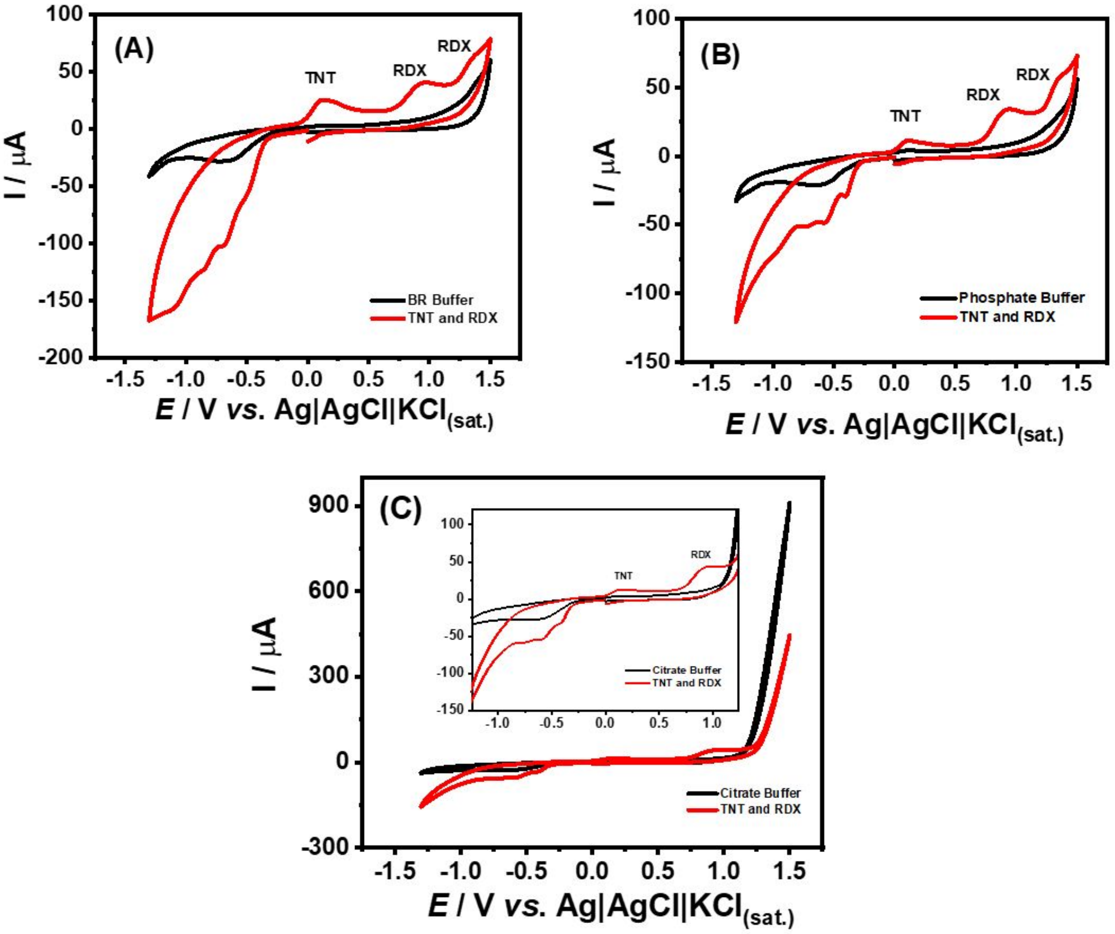

Figure S7. Cyclic voltammograms recorded in different supporting electrolytes at pH 6.0 in the presence of TNT 0.1 mmol L^{-1} and RDX 1 mmol L^{-1}; **(A)** BR, **(B)** Phosphate and **(C)** Citrate buffers. Conditions: step: -5 mV, v = 50 mV s^{-1}.

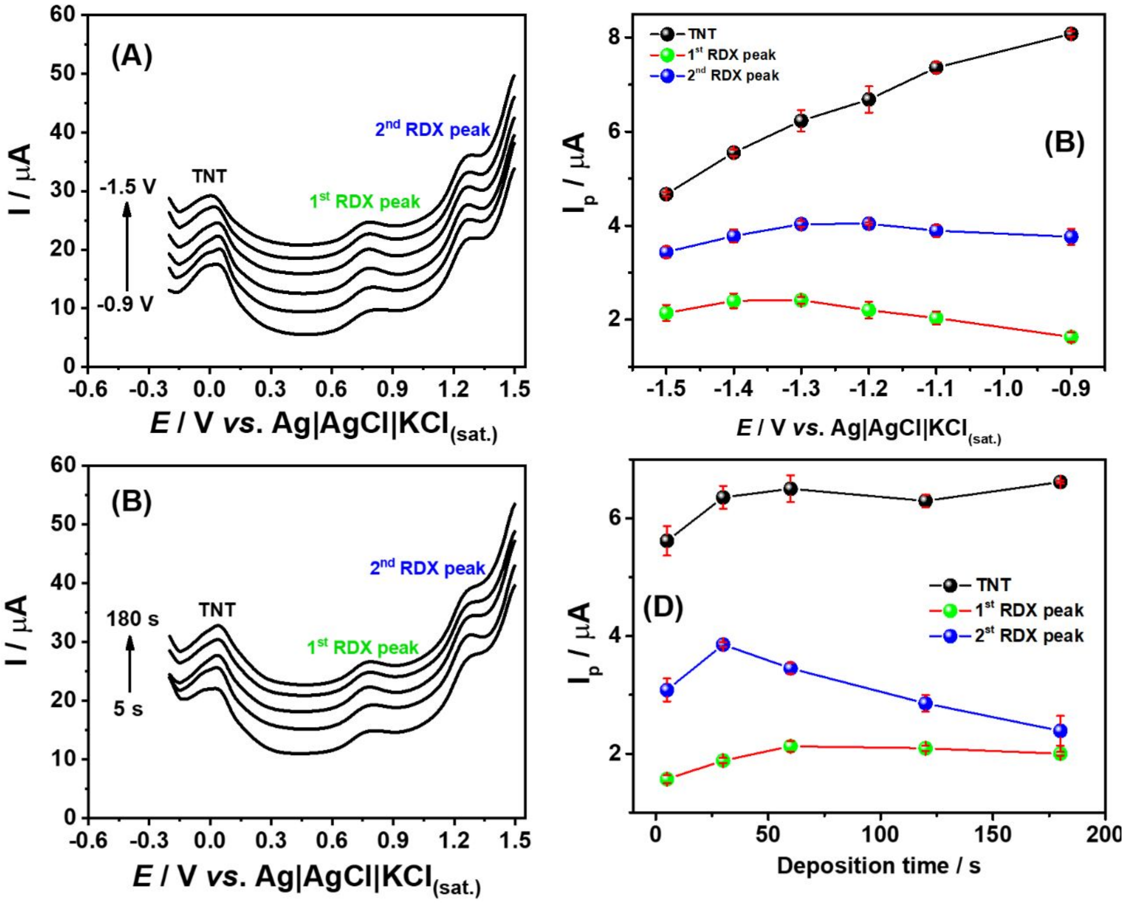

Figure S8. Differential pulse voltammograms recorded in 0.12 mol L^{-1} BR buffer (pH 6.0) varying the applied potential (-0.9 V to -1.5, Figures A and B) and deposition time (5 to 180 s, Figures C and D); in the presence of 25 μmol L^{-1} TNT and 200 μmol L^{-1} RDX. **DPV parameters:** potential step potential = 5 mV, amplitude = 70 mV, modulation time = 40 ms, v = 10 mV/s.

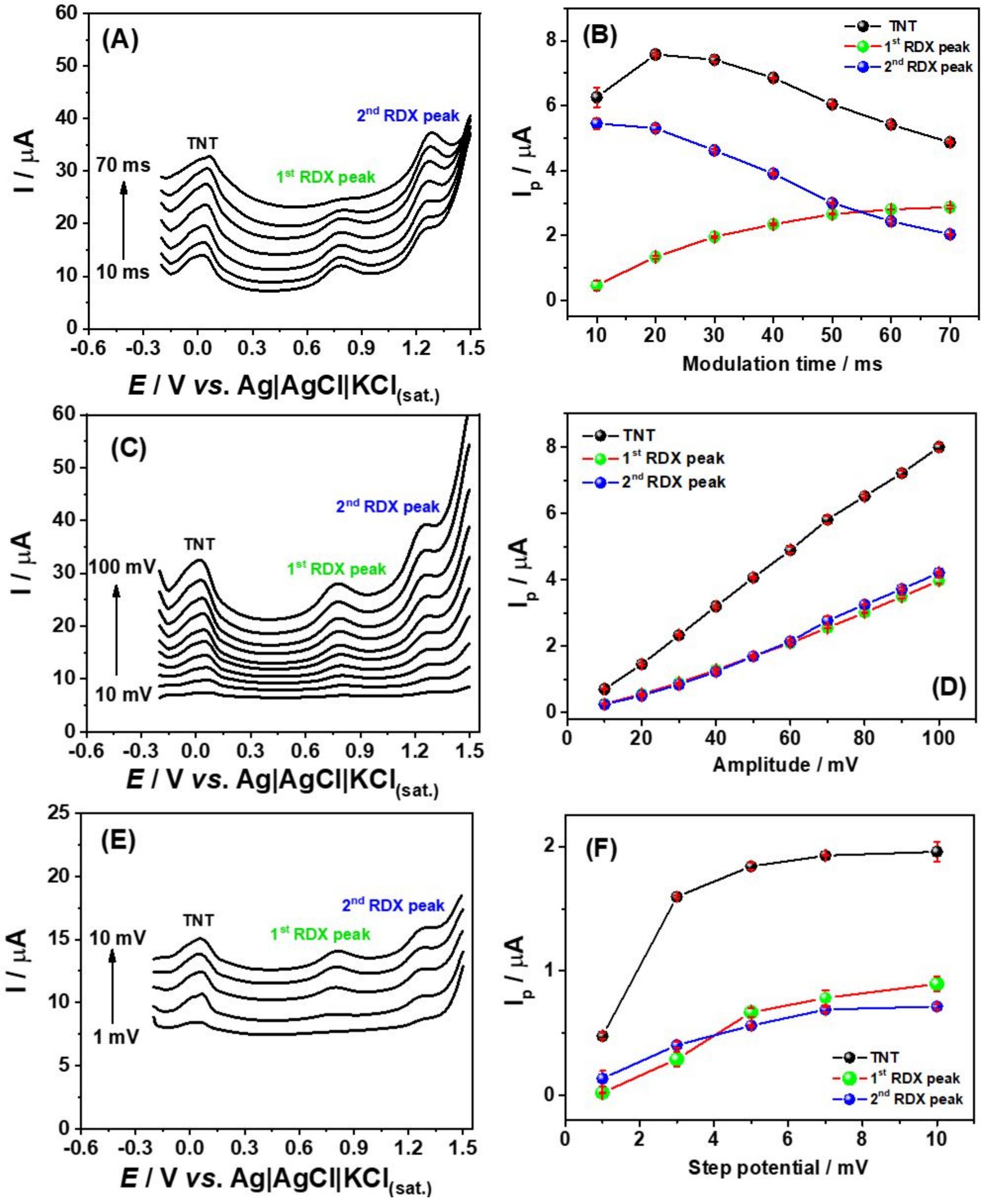

Figure S9. Differential pulse voltammograms recorded at BR buffer (pH 6.0) varying the modulation time (10–70 ms, Figures A and B), amplitude (10–100 mV, Figures C and D), and step potential (1–10 mV, Figures E and F); in the presence of 25 μmol L^{-1} TNT and 200 μmol L^{-1} RDX. Other conditions: Applied potential -1.2 V (*vs.* Ag|AgCl|$KCl_{(sat.)}$), deposition time 60 s.

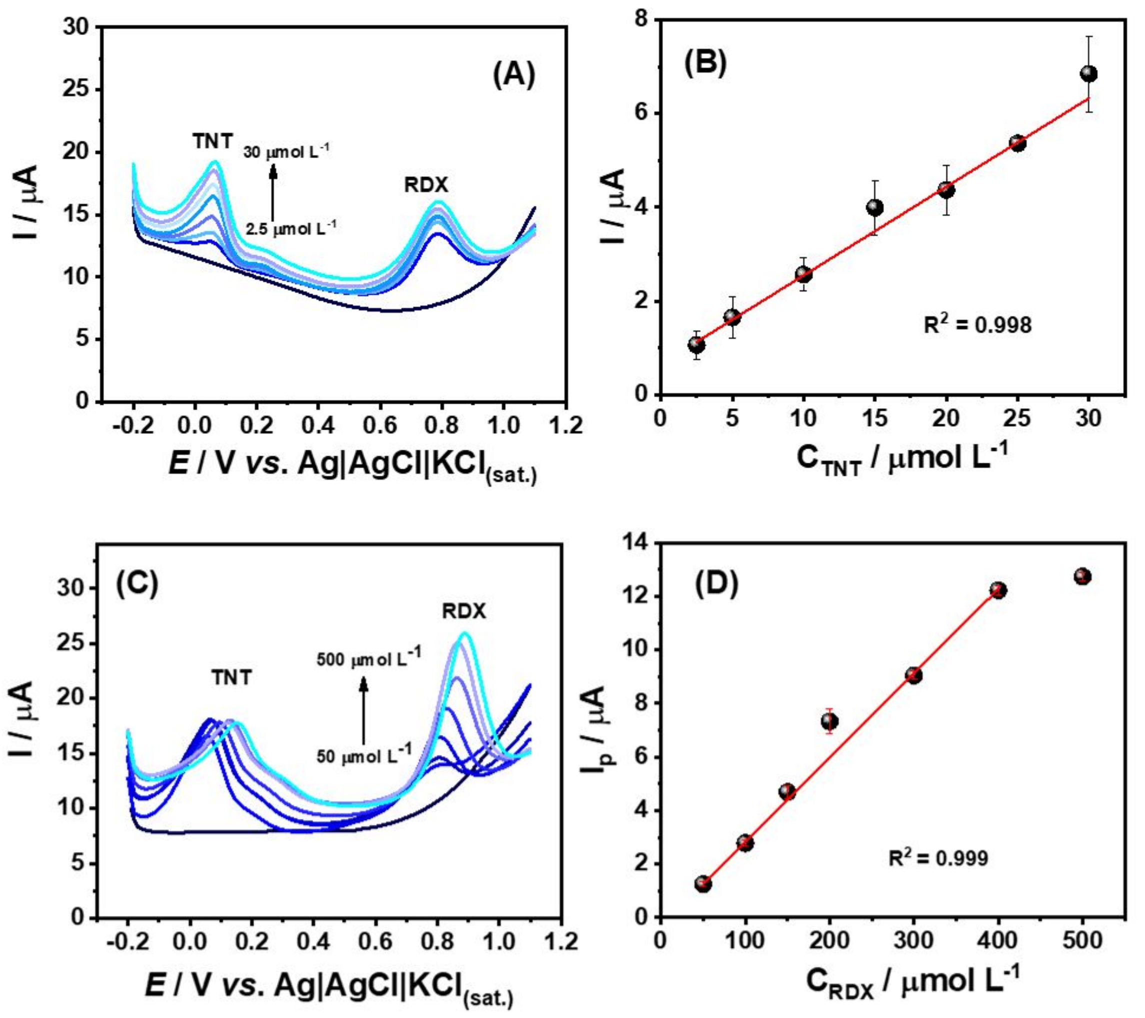

Figure S10. Differential pulse voltammogram recorded in 0.12 mol L^{-1} BR buffer (pH 6.0, black lines) at increasing TNT concentrations (2.5 - 30 µmol L^{-1}) fixing 200 µmol L^{-1} RDX in Figures A and B, and at increasing RDX concentrations (50 - 500 µmol L^{-1}) fixing 15 µmol L^{-1} TNT in Figures C and D. Applied potential -1.3 V, deposition time 30 s. DPSV parameters: step potential = 5 mV, amplitude = 100 mV, modulation time 40 ms, ν = 10 mV/s.

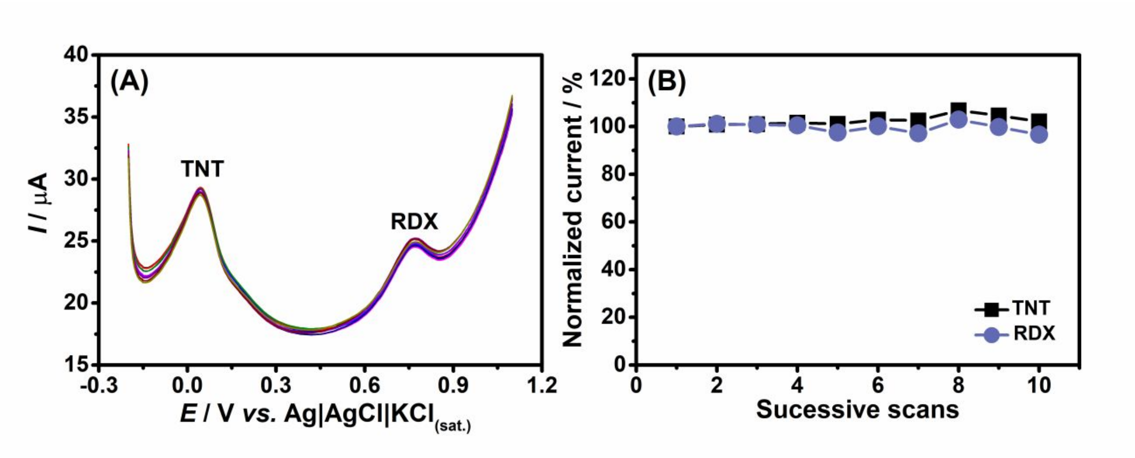

Figure S11. (A) DPV measurements obtained from ten successive scans (n = 10) in the presence of 20 μmol L⁻¹ TNT and 100 μmol L⁻¹ RDX; **(B)** Corresponding variation in current intensities. Applied potential -1.3 V, deposition time 30 s. DPV conditions: modulation time = 40 ms; step potential = 5 mV and amplitude = 100 mV.

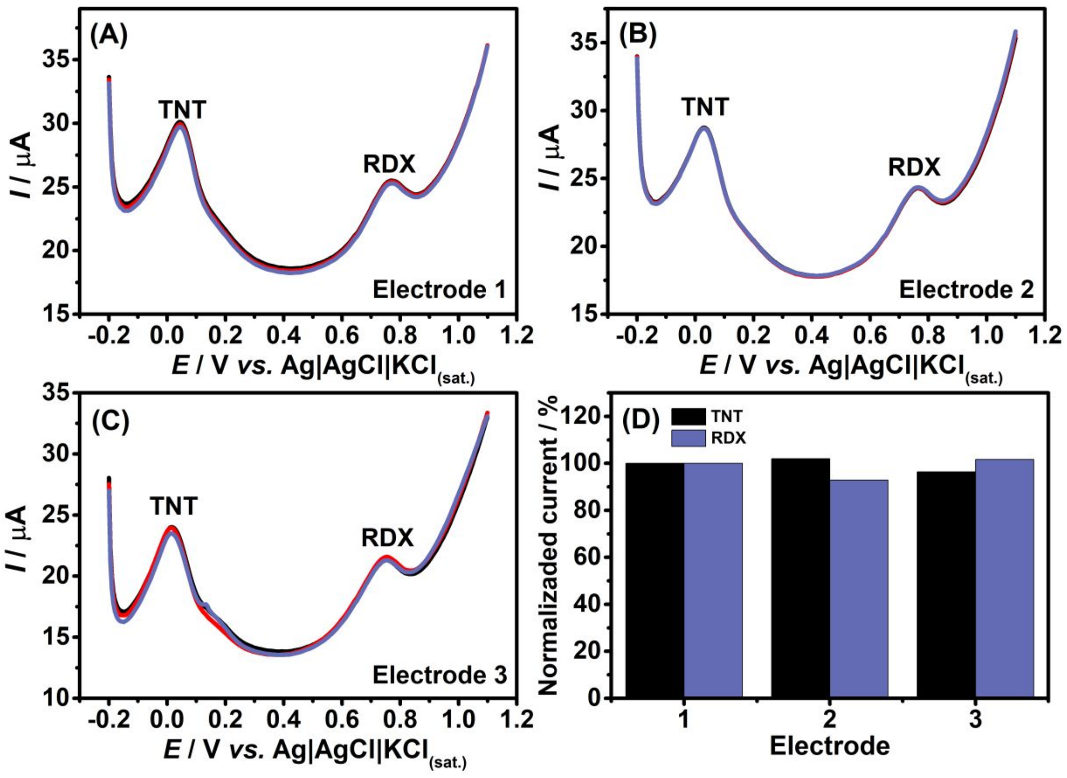

Figure S12. (A,B,C) DPV measurements obtained from three successive scans (n = 3) on three different electrodes in the presence of 20 μmol L^{-1} TNT and 100 μmol L^{-1} RDX; **(D)** Corresponding variation in current intensities. Applied potential -1.3 V, deposition time 30 s. DPV conditions: modulation time = 40 ms; step potential = 5 mV and amplitude = 100 mV.

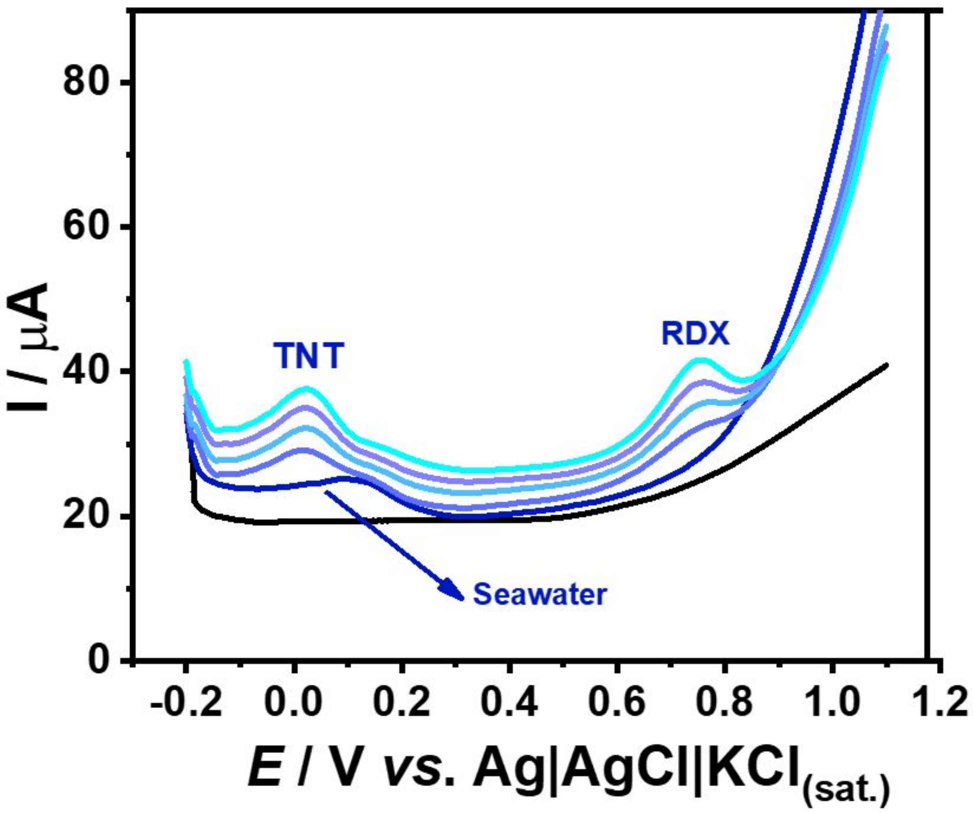

Figure S13. Differential pulse voltammogram responses for the analysis of seawater samples (dark blue line) and successive additions of increasing concentrations of TNT (10, 15, 20 and 25 µmol L^{-1}) and RDX (100, 150, 200 and 250 µmol L^{-1}). The black lines refer only to the blank signal (BR buffer pH 6.0). Applied potential -1.3 V, deposition time 30 s. DPSV parameters: step potential = 5 mV, amplitude = 100 mV, modulation time 40 ms, v = 10 mV/s.
